# Z-Nucleic Acid-Binding Protein (ZBP)1 (DAI/DLM1) Regulation in Antiviral Cell Death Pathways

**DOI:** 10.3390/pathogens15090972

**Published:** 2026-09-14

**Authors:** Edward S. Mocarski, Hongyan Guo

**Affiliations:** 1 Department of Microbiology & Immunology, Stanford Medical School, Stanford University, Stanford, CA 94305, USA; 2 Department of Microbiology & Immunology, Emory Medical School, Emory Vaccine Center, Emory University, Atlanta, GA 30322, USA; 3 Department of Microbiology & Immunology, Louisiana State University Health Sciences Center, Shreveport, LA 71103, USA

**Keywords:** herpesvirus, poxvirus, influenza, immune modulation, pathogenesis, inflammation

## Abstract

Metazoan organisms employ an array of overlapping innate and adaptive immune mechanisms to defend against infection and prevent disease. Z-nucleic acid-binding protein (ZBP)1 (also denoted DAI or DLM1) has emerged as a key nucleic acid (NA) sensor within mammalian cells responding to stress, infection or inflammation. Once engaged and activated by Z-nucleic acid, ZBP1 recruits receptor-interacting protein (RIP) kinase (RIPK)3 through RIP homotypic interaction motif (RHIM)-mediated interactions to promote regulated cell death and inflammatory signaling, with necroptosis representing a particularly important antiviral defense mechanism. DNA viruses have coevolved with their hosts to encode diverse inhibitors that suppress ZBP1-RIPK3 signaling as well as other cell death pathways, highlighting the strong selective pressure imposed by necroptosis on viral replication. In contrast, RNA viruses lack inhibitors and activate multiple interconnected death pathways, including necroptosis, apoptosis, and pyroptosis. Increasing evidence indicates that both viral and cellular RNA transcripts adopt Z-conformation and activate ZBP1. Recent studies further implicate stress-induced transcriptional read-through, called disruption of transcription termination, of endogenous retroviral elements as important endogenous sources of Z-RNAs. These findings establish ZBP1 as a central sensor linking aberrant nucleic acid structures to RIPK3-dependent cell death and inflammation during viral infection and cellular stress of disease processes.

## 1. Introduction

Intracellular pathogens, including many viruses, encode immunomodulatory gene products to subvert host defenses in order to replicate, disseminate and cause disease [1,2,3]. The regulated cell death (RCD) pathways, apoptosis, necroptosis and pyroptosis, provide first-line defense against intracellular pathogens [4,5,6,7,8]. Cell intrinsic signaling via preformed sensors and signaling adaptors initiates the altruistic self-sacrifice of infected cells to cut short production and reduce spread of infectious progeny as well as the consequent inflammatory tissue damage. Intrinsic (mitochondrial) apoptosis represents an ancient, widely conserved pathway for removing excess cells during embryonic development [9] and for tissue homeostasis throughout life [10], generally proceeding without inflammatory consequences [11,12,13]. Extrinsic apoptosis initiated by caspase-8 (CASP8) represents a second pathway that is dependent on both death effector domain (DED) dimerization and death domain (DD) recruitment of signaling adaptor, Fas-associated via DD (FADD) to form a death-inducing signaling complex (DISC) and TNF family death receptors (TNFR1, Fas or TRAIL) resulting in complex II (FADD-CASP8-RIPK1). This complex may be subverted by dedicated mammalian DNA virus-encoded inhibitors [4,5,7] and is now known to form in a variety of other settings where these preformed signaling adaptors assemble downstream of innate pattern recognition and immune receptors that are regulated by cellular FLICE-like inhibitory protein (cFLIP), RIPK1, polyubiquitinylation of core components and participation of additional adaptors including those associated with inflammasome signal transduction [14,15,16,17,18,19,20,21]. Over millions of years, DNA viruses such as cytomegaloviruses have evolved and adapted to counter such host defense mechanisms [8]. In this review, we will focus on the contribution of ZBP1 recognition of virus-encoded as well as host cell-encoded Z-form NA and the viral suppression of cell death-dependent as well as cell death-independent signaling.

Z-nucleic acid-binding protein (ZBP)1 (also denoted DAI or DLM1) functions as a key nucleic acid (NA) sensor within mammalian cells responding to stress, infection or inflammation [8,22,23,24]. ZBP1 recognizes Z-form nucleic acid (NA) through two copies of a winged helix-turn-helix Zα motif. Although most early work focused on Z-DNA, the predominant ZBP1 ligand recognized in natural settings is Z-RNA derived either from viral or cellular mRNA transcripts [8,25,26]. Once engaged and activated, ZBP1 recruits receptor-interacting protein (RIP) kinase (RIPK)3 through RIP homotypic interaction motif (RHIM)-mediated interactions. ZBP1-dependent activation of RIPK3 triggers alternate programmed cell death modes: (i) necroptosis via RIPK3-mediated phosphorylation of MLKL resulting in cell leakage; (ii) caspase-8-dependent apoptosis following RIPK3 RHIM-mediated recruitment of RIPK1 followed by RIPK1 death domain recruitment of Fas-associated protein with death domain (FADD); and, (iii) inflammatory signaling via inflammasome activation leading to gasdermin-dependent pyroptosis. Together with mitochondrial (intrinsic) apoptosis, these cell death pathways may halt viral infection by eliminating host cells. All are subverted by dedicated cell death inhibitors encoded by large DNA viruses as illustrated by cytomegaloviruses native to humans and mice [27]. Herpesviruses and poxviruses lacking necroptosis suppressors are highly attenuated, revealing necroptosis as the predominant regulated cell death pathway that eliminates infected cells before viral progeny can be produced [8]. Virus-induced necroptosis apparently evolves as an alternate host defense pathway unleashes when caspase-8 becomes compromised, occurring through the action of DNA virus-encoded caspase inhibitors. Two well established modulation mechanisms prevent unleashing necroptosis during DNA virus infection: (i) a poxvirus-encoded competitor that prevents ZBP1 Zα-dependent recognition of Z-RNA and (ii) a herpesviruses-encoded RHIM competitor that prevents recruitment of RIPK3 as well as RIPK1. RNA viruses such as influenza A virus do not encode inhibitors and, as a result, trigger the full manifestation of RIPK3-mediated cell death (necroptosis, apoptosis, pyroptosis, referred to as PANoptosis) with necroptosis dominating [28,29]. Z-RNA originates from viral mRNA as well as from cellular transcripts accumulating early during infection. In both noninfectious settings as well as during influenza or herpesvirus infection, ZBP1-RIPK3-MLKL necroptosis becomes triggered as a consequence of stress-induced self-complementary transcripts that arise from disruption of transcription termination (DoTT), particularly involving endogenous retroviral elements [26]. Thus, transcriptional read-through and production of self-complementary RNA contribute to accumulation of Z-RNAs that activate ZBP1.

## 2. Regulated Cell Death in Host Defense

RCD pathways provide a critical contribution to the elimination of virus-infected cells, preventing wider tissue damage as well as pathogen dissemination and disease. Cell death provides important host control during the innate as well as the adaptive phases of the host immune response to virus infection that ultimately clears active viral infection. A contribution of intrinsic apoptosis to immune control has been long recognized across the spectrum of multicellular organisms [4,5,6,7]. In mammals, apoptosis contributes to elimination of stressed and damaged cells in both infectious and noninfectious disease settings [30,31]. The natural mouse pathogen murine cytomegalovirus (MCMV) has provided key insights into the contribution of individual cell death pathways to antiviral immunity and the pathogen–host balance [8,27]. MCMV deploys specific virus-encoded suppressors of intrinsic apoptosis [32,33,34,35], CASP8-dependent (extrinsic) apoptosis [33,36,37] and receptor-interacting protein (RIP) kinase (RIPK) homotypic interaction motif (RHIM) signaling. The inhibitors of intrinsic apoptosis and CASP8-mediated apoptosis are the only immunomodulatory functions conserved over evolution in both rodent and primate cytomegaloviruses. The RHIM signaling inhibitor has not been conserved in primate cytomegaloviruses but is employed by primate alphaherpesviruses such as herpes simplex virus (HSV) [8]. Studies of MCMV cell death suppressor mutants reveal the relative value of intrinsic and extrinsic apoptosis as well as necroptosis mechanisms in controlling virus infection [38]. MCMV studies also highlight the importance of CASP8 directly cleaving CASP3 independent of mitochondrial steps in the TNF response [30,39,40,41]. Activation of mitochondrial proapoptotic BCL2 family members BAX and BAK is blocked by dedicated MCMV-encoded inhibitors [4,5,8,34,35,42,43]. Importantly, although apoptosis may be viewed as noninflammatory during development [20,21], both intrinsic and extrinsic apoptosis contribute to inflammation in infectious disease settings [44], including MCMV infection within its natural mouse host [30,41] as well as following bacterial lipopolysaccharide-induced shock [45].

Necrotic forms of RCD (necroptosis and pyroptosis) contribute to host defense by killing cells through the formation of pores allowing consequent leakage of cell contents [20,21,46]. The release of damage-induced molecular patterns (DAMPs) contributes to inflammatory outcomes in wide-ranging disease settings [47]. RIPK1-mediated activation of RIPK3 drives necroptosis downstream of TNF family death receptors, particularly when proapoptotic CASP8 protease activity is compromised [48,49,50]. Necroptosis may be unleashed through the impact of MCMV-encoded CASP8 inhibitors [51] as well as by caspase inhibitors encoded by other herpesviruses such as herpes simplex virus (HSV) [52] and poxviruses such as vaccinia [53]. Prompted by this dramatic unleashing and potentiation of necroptosis by virus-encoded CASP8 inhibitors, Kaiser et al. [51] discovered that necroptotic signaling is responsible for the striking midgestational failure of CASP8-deficient mice [54]. Phenotypes of CASP8-deficient mice as well as germline deficiency in FADD [55] had suggested a role for extrinsic apoptosis in development even though deficiency in TNF family ligands or DD receptors never phenocopy developmental defects revealed in either CASP8 or FADD deficiency [56,57]. Once rescued through genetic crosses that attributed developmental failure to RIPK1-RIPK3-mediated necroptosis [51,58,59,60], two seismic shifts occurred in the cell death field: (1) extrinsic apoptosis and necroptosis were shown to be dispensable for full development of fertile and otherwise immunocompetent mice that retain sufficient innate and adaptive immune competence to be able to control viral infection [51,61] and (2) the stage was set to dissect the distinct contributions of both CASP8- and RIPK3-regulated signaling pathways to host defense and inflammatory disease [8,30].

Importantly, despite markers of additional cell death signaling that have been called PANoptosis, necroptosis predominates in eliminating infected cells and becomes particularly potent when CASP8 is inhibited [28]. Because its natural host is the laboratory mouse, the natural mouse pathogen MCMV has been instrumental in comparative studies revealing the importance of necroptosis compared to extrinsic and intrinsic apoptosis in host defense determinant that impacts viral pathogenesis [8,27,30,38,41]. Combination of pathway-specific cell death suppressor mutant viruses and mutant mouse strains has opened the way to systematic understanding of cell death-mediated host control over virus infection and emphasizes the importance of necroptosis over other death pathways.

## 3. Z-NA Sensing by ZBP1

**Figure 1:**
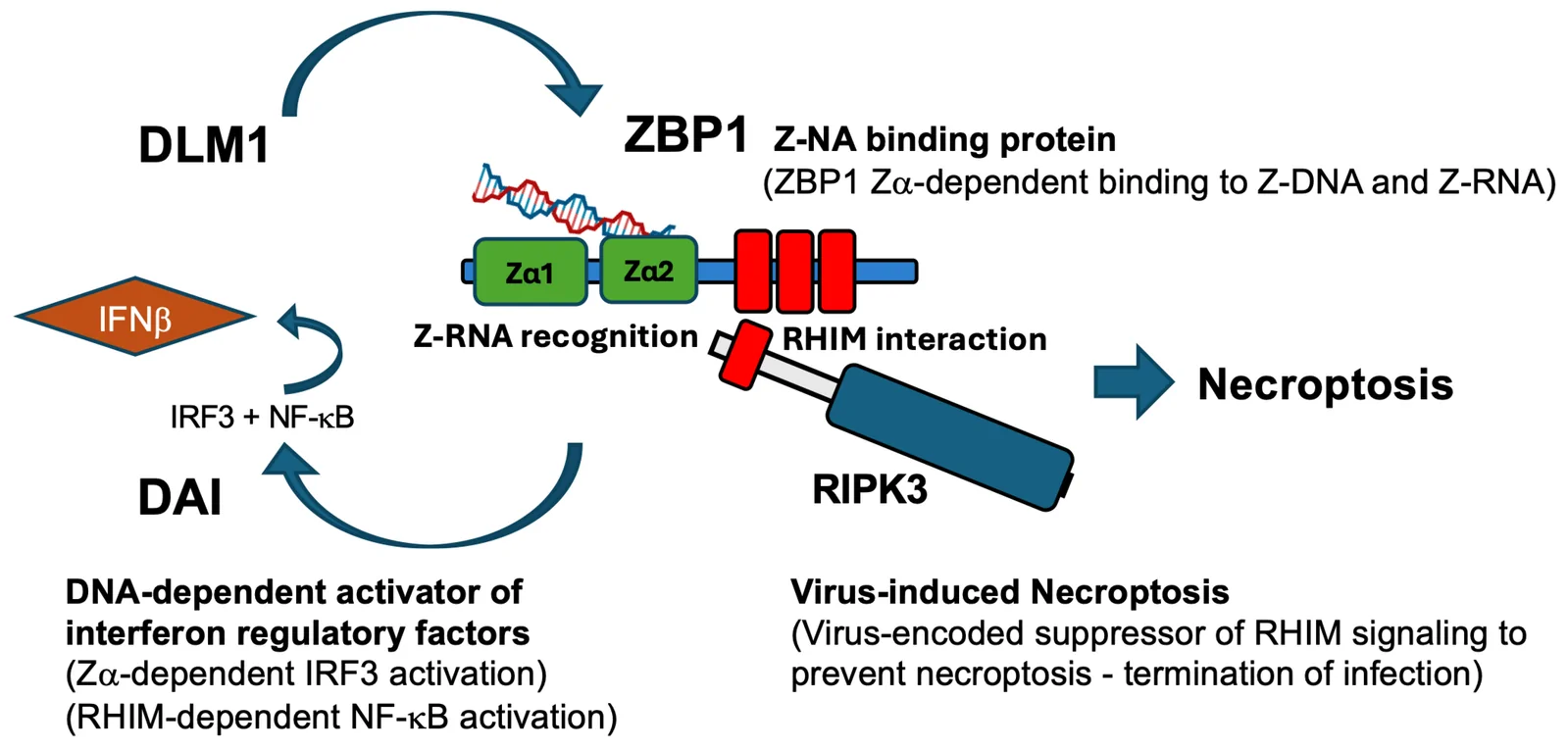
**ZBP1/DAI/DLM1 links Z-nucleic acid sensing to RIPK3-dependent necroptosis.** ZBP1 was initially identified as a prominent IFN-inducible protein DLM1 [62] and later characterized as DAI, a DNA-induced activator of interferon regulatory factors that promotes type I IFNβ induction through activation of IRF3 and NF-kB [69] (as depicted by the curving arrows). Priorstudies established ZBP1 as a Z-NA-binding protein containing two N-terminal Zα domains, Zα1 and Zα2, that recognize Z-form DNA or RNA, which were later shown to trigger C-terminal RHIMs (red rectangles) to recruit RIPK3 and other RHIM-containing signaling proteins [8]. RIPK3 is composed of an N-terminal protein kinase domain (teal rectangle) and a C-terminal RHIM (red rectangle). Thus, upon sensing Z-form NA, ZBP1 recruits RIPK3 through RHIM-dependent interactions to initiate necroptosis dependent on protein kinase activity [18,25,27,43] in a manner first described for RIPK1 recruitment of RIPK3 [48,49,50].

First recognized as an abundant interferon (IFN)-stimulated gene (ISG) product in human and mouse cells [62], ZBP1 and adenosine deaminase acting on RNA (ADAR), an mRNA editing enzyme conserved across all metazoans [63,64], emerged as defining members of the Z-nucleic acid (NA) binding protein family [8,22]. This family is defined by common winged helix-turn-helix Zα domains long known to bind left-handed double-stranded DNA or RNA [65,66,67,68]. ADAR and ZBP1 exhibit interrelated signaling dependencies that continue to provide interesting insights into RNA modification and sensing [22,23,24]. ZBP1 contains two Zα motifs as well as three iterations of a RHIM that together coordinate recognition of Z-NA and recruitment of RIPK3 [69] as well as other RHIM-containing proteins, RIPK1, Toll and interleukin-1 receptor-containing adaptor-inducing interferon b (TRIF, also called TICAM1). Even though RIPK1 and TRIF are conserved broadly and functional across vertebrate species, ZBP1 is not conserved but exhibits evolutionary variability across mammals, reptiles, amphibians and cartilaginous fish [70]. ZBP1 along with necroptosis signaling adaptor RIPK3 and executioner mixed lineage kinase domain-like (MLKL) are only conserved in certain vertebrate lineages [70,71,72,73,74,75]. Importantly, both rodents and primates, including humans, conserve the full spectrum of necroptosis signaling components and mechanisms (depicted in Figure 1).

**Figure 2:**
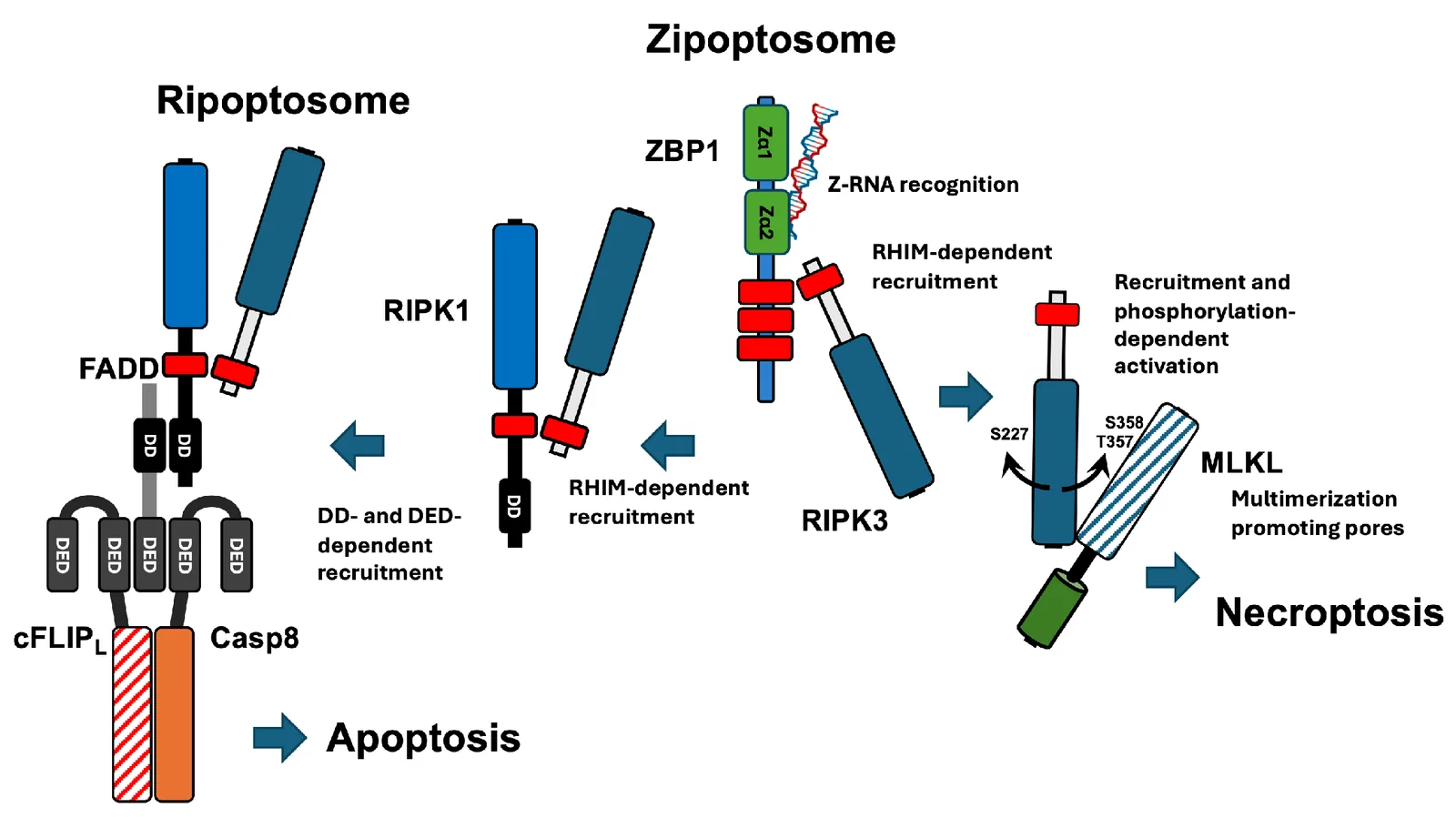
**ZBP1-driven cell death signaling complex formation**. Upon recognition of Z-form RNA through its Zα domains, ZBP1 recruits RIPK3 via RHIM-dependent interactions to form a ZBP1-containing signaling platform, referred to as the Zipoptosome (other terms used: PANoptosome or noncanonical necrosome). This complex can promote distinct downstream cell death outcomes depending on the cellular context, presence of inhibitors and availability of signaling components. RHIM-mediated recruitment of RIPK1, followed by death domain (DD) and death effector domain (DED) recruitment of FADD, CASP8, and cFLIP results in assembly of a Ripoptosome-like complex that drives apoptosis dependent on the CASP8 protease domain (orange rectangle) [14,17,25,81]. In the presence of virus-encoded CASP8 inhibitors, ZBP1-associated RIPK3 activation promotes formation of the necrosome, leading to RIPK3-mediated recruitment and phosphorylation of itself as well as the C-terminal pseudokinase domain (hatched teal) of MLKL and execution of necroptosis mediated by the N-terminal helical bundle (green barrel) of MLKL [8,23,25,26,92,93]. In settings where necroptosis, apoptosis, and inflammasome activation are coordinated, this broader series of ZBP1-dependent platforms have been termed a PANoptisome [81].

ZBP1 is both a type I IFN stimulated gene product and acts as a pathogen sensor in rodents and humans to nitiate RIPK3-dependent signaling with at least three potential outcomes: (1) activation of RIPK3 drives necroptosis via MLKL-dependent pore formation, (2) apoptosis by recruiting RIPK1, FADD and CASP8 and (3) inflammasome activation via CASP1 leading to tissue injury and disease pathogenesis [18,19,76,77]. Even broader cell death-independent inflammatory signaling outcomes are also observed [78,79,80]. This is all achieved through the formation of a RHIM-dependent complex referred to as a Zipoptosome [8] or PANoptosome [81] analogous to the Ripoptosome formed via RIPK1 recruitment of RIPK3 (Figure 2). Activation of ZBP1 by Z-NA in natural settings was discovered [82,83,84,85,86,87,88] and subjected to detailed mechanistic evaluation [88,89,90,91] through herpesvirus- and poxvirus-encoded necroptosis inhibitors targeting ZBP1 and RIPK3 [8,23,25,26,92,93].

**Figure 3:**
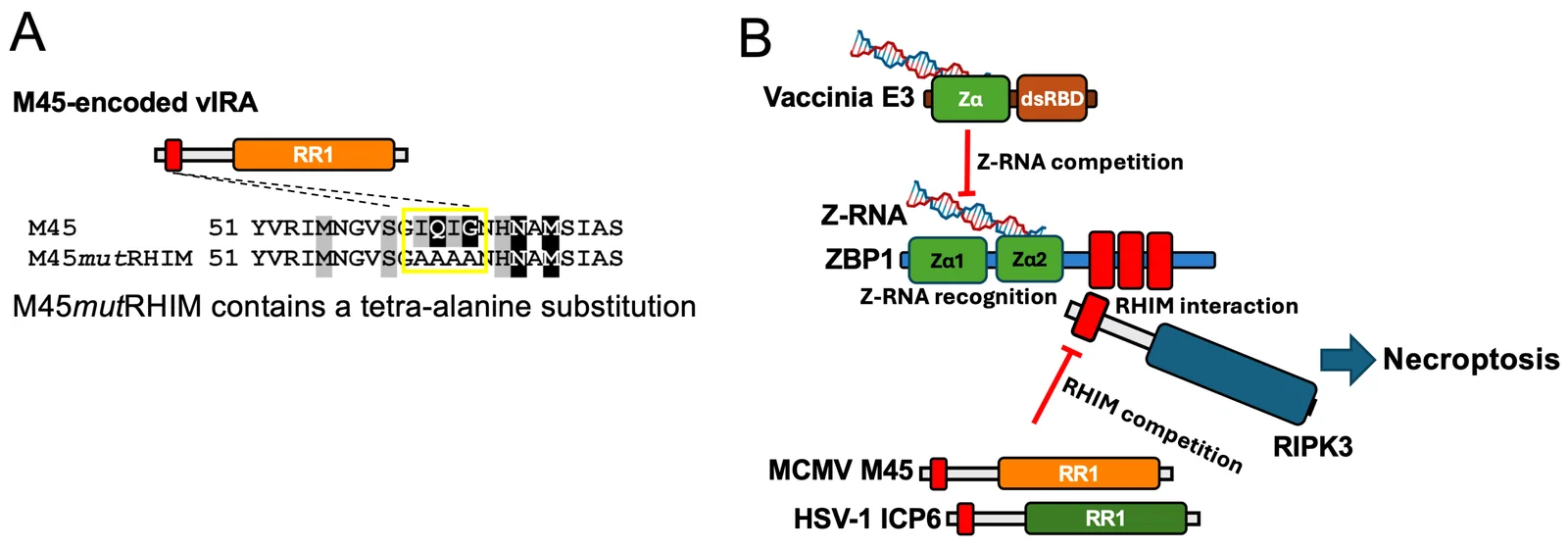
**Viral suppression of ZBP1-driven necroptosis.** (**A**,**B**) Large DNA viruses encode dedicated inhibitors that block ZBP1-dependent activation of RIPK3 and necroptosis. The murine cytomegalovirus M45-encoded viral inhibitor of RIP activation (vIRA) contains a RHIM within its ribonucleotide reductase 1 (RR1)-like region; mutation of the core RHIM sequence to alanines disrupts this inhibitory activity and permits ZBP1–RIPK3 signaling (**A**). Poxvirus E3 suppresses ZBP1 activation through its N-terminal Zα domain, which competes for Z-form nucleic acid-binding in cooperation with its C-terminal double stranded RNA binding domain (dsRBD) in order to prevent ZBP1 engagement of Z-RNA. In contrast, herpesvirus RHIM-containing proteins, including MCMV M45 (viral inhibitor of CASP8 activation) and HSV1 ICP6 proteins, interfere downstream by competing with RHIM-mediated assembly of the ZBP1–RIPK3 complex (**B**) [8].

The specialized pathogen sensor role of ZBP1 in triggering virus-induced necroptosis [8,25] emerged from studies of cell death inhibitors encoded by the herpesviruses MCMV and HSV1 as well as the poxvirus and vaccinia [8,94]. In these mutant DNA virus settings as well as during infection with influenza and other RNA viruses, including coronaviruses, ZBP1 senses Z-RNA [25,88,89,90,91,95,96,97,98,99]. RNA viruses are not known to encode dedicated necroptosis inhibitors. The importance of ZBP1 recruitment of RIPK3 and induction of necroptosis first emerged from studies with mutant MCMV carrying a four-amino-acid substitution within the RHIM signaling inhibitor M45 (M45mutRHIM, Figure 3A) that revealed a pattern of RIPK1-independent necroptosis during infection of mouse cells and mice [84,85].

Although now understood to be responsible for virus-induced necroptosis, the pathogen sensing potential of ZBP1 initially emerged from studies focused on induction of a type I IFN response following transfection of plasmid DNA or infection with HSV1 [100,101]. ZBP1 was denoted DNA-induced activator of IFN regulatory factors (DAI) due to an ability to mediate TANK-binding kinase (TBK)1 phosphorylation of IFN regulatory factor (IRF)3. Soon after this report, RHIM-dependent ZBP1 recruitment of RIPK1 was revealed to control induction of the transcription factor NF-kB [69] that functioned in concert with IRF3 to drive induction of IFNβ. Although these observations were reproducible, ZBP1-mediated type I IFN induction is largely redundant and dispensable for sensing DNA in human or mouse cells and mice [102]. The broadly conserved pathogen sensing pathways in metazoans came to light [103], most prominently cGAS-STING [104,105,106,107] recognition of cytosolic DNA to trigger TBK1-IRF3-mediated induction of type I IFN and interferon-stimulated genes (ISGs). As studies proceeded, early observations on ZBP1 as a sensor gave way to now well-documented focus on ZBP1 cell death and inflammatory signaling mediated through RIPK3 [8].

RIPK1 remains an intensively studied RHIM-containing adaptor protein kinase contributing to NF-kB activation along with a DD mediating recruitment of FADD to assemble RIPK1-FADD-CASP8-cFLIP FADDosome (complex II) mediating extrinsic apoptosis and alternate RHIM-dependent recruitment of RIPK3 to initiate necroptosis, activities that are regulated by polyubiquitylation, autophosphorylation and cleavage of RIPK1 [108,109] as well as ZBP1 and other adaptors [110]. When kinase activity is blocked by small-molecule kinase inhibitors or mutagenesis, RIPK3 mediates recruitment of RIPK1 via RHIM–RHIM interactions, facilitating formation of the Ripoptosome, a FADDosome/complex II with RIPK3, and the execution of apoptosis [111,112]. TRIF, the RHIM adaptor involved in TLR3 and TLR4 induction of type I IFNs via TBK1 and NF-kB, also mediates alternate necroptosis and apoptosis outcomes by rrecruitng RIPK3 [113,114,115].

**Figure 4:**
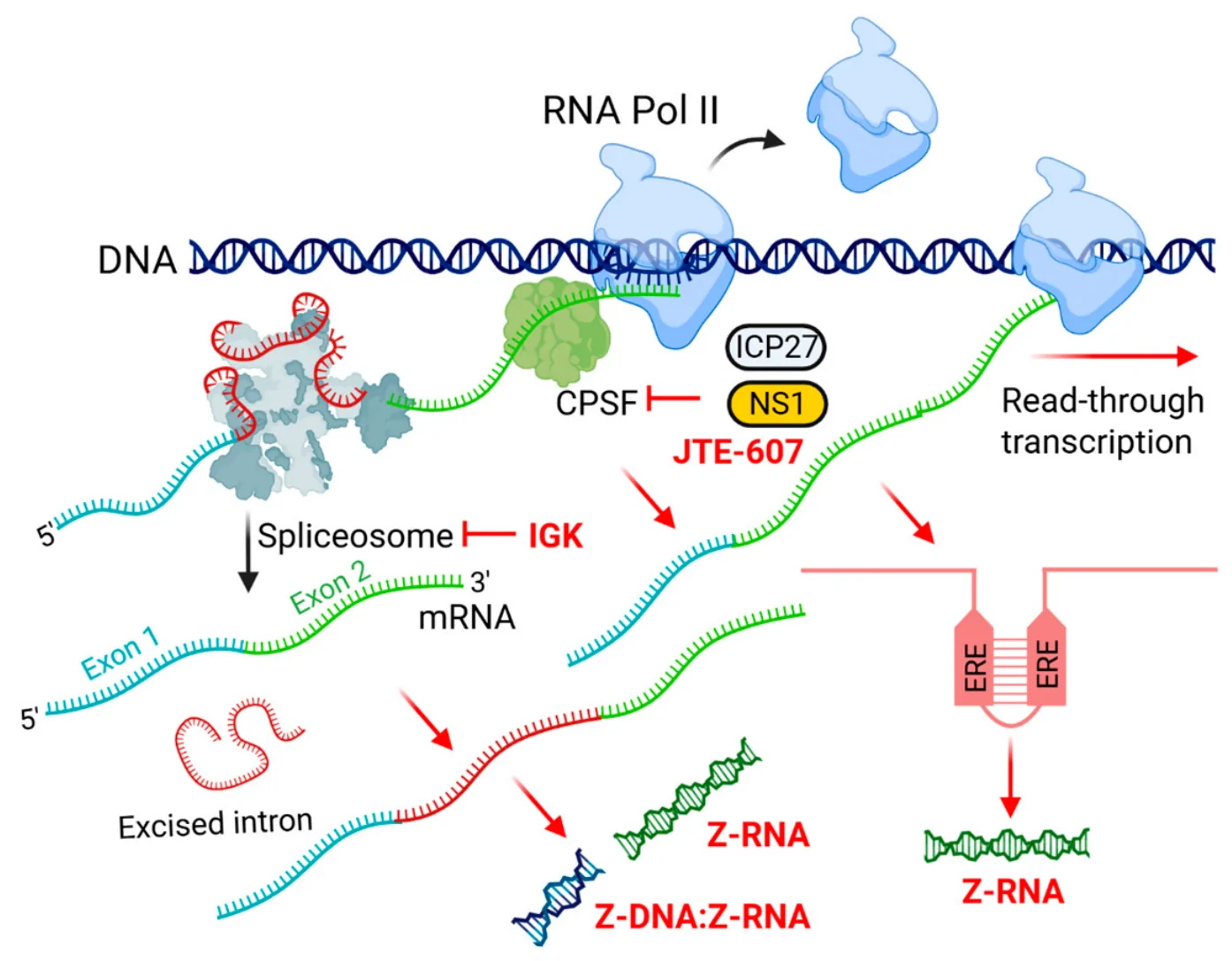
**Generation of Z-NA ligands.** Schematic model illustrating potential sources of Z-form NA generated during RNA polymerase II-dependent transcription and RNA processing [26]. Nascent pre-mRNAs are normally processed by the spliceosome to remove introns and by cleavage/polyadenylation factors, including CPSF, to terminate transcription and generate mature mRNAs. Disruption of these processes may promote accumulation of aberrant RNA species capable of adopting Z-conformations. Inhibition of splicing, shown here with the spliceosome inhibitor IGK, may increase intron-derived RNA species. Inhibition of CPSF-dependent 3′-end processing by viral factors such as HSV-1 ICP27 or influenza virus NS1, or by the small-molecule inhibitor JTE-607, may induce read-through transcription. Extended read-through transcripts incorporate self-complementary, endogenous retroelement-derived sequences and form Z-RNA or Z-DNA:Z-RNA hybrid structures, providing ligands for Z-form NA sensor ZBP1. Created in BioRender. Guo, H. (2026) https://BioRender.com/q7h7fwm.

Full-length ZBP1 localizes to the cytoplasm dependent on its carboxyl-terminal signaling domain [116] and central RHIM-containing region [69], where sensing of Z-RNA by N-terminal Zα domains early in virus infection triggers necroptosis [88,89,90,95,97]. Studies on the RNA virus influenza initially revealed Z-RNA triggers ZBP1 to drive a predominant pattern of virus-induced necroptosis [25,28,97]. Recent work has further refined the identity of physiological ZBP1 ligands during virus infection [26]. Although Z-RNAs detected during HSV1 and influenza A virus infection were initially presumed to derive primarily from viral transcripts, transcriptome-wide Z-NA mapping now indicates that aberrant host-cell transcripts are major, and in some settings sufficient, activators of ZBP1. These host-derived Z-RNAs arise when virus infection disrupts normal transcription termination, producing unusually long 3′ extensions of cellular mRNAs enriched for intergenic endogenous retroelement sequences. In HSV1 infection, the viral immediate-early protein ICP27 contributes to this disruption of transcription termination (DoTT) and influenza A virus NS1 promotes a similar outcome as depicted in Figure 4 [26]. Thus, ZBP1 activation during infection reflects not only recognition of pathogen-encoded nucleic acids but also detection of infection-induced host transcriptional stress. This model broadens ZBP1 from a conventional pathogen sensor to a sentinel of pathological Z-RNA accumulation generated by viral DoTT that directly alters the physical characteristics of accumulating host mRNA.

Recently, the relationship between ZBP1 sensing of Z-RNA and ADAR1-mediated modification of mRNA has come to light [24,92] such that ADAR1 naturally prevents recognition by endogenous cellular retroelement transcripts that exhibit double-stranded RNA due to the sequence characteristics of long inverted repeats (LTRs) that take on a Z-NA conformation [117,118,119]. In addition to ADAR1-mediated suppression of endogenous Z-RNA, studies have identified pre-mRNA splicing as an additional layer of protection against inappropriate ZBP1 activation. Spliceosome dysfunction, including pharmacological inhibition or disruption of core spliceosomal components, promotes accumulation of Z-NA and triggers ZBP1-dependent cell death. Spliceosome inhibition induces the accumulation of Z-RNA being exported from the nucleus to the cytoplasm, where ZBP1 is engaged and initiates downstream death signaling [120]. Spliceosome stress may also generate Z-RNA:DNA hybrids, linking defective RNA processing, R-loop biology, and ZBP1 activation [121]. Together with the ADAR1 literature, these findings support a model in which multiple RNA-surveillance pathways normally restrain endogenous Z-RNA accumulation. When RNA editing, splicing, transcription termination, or nuclear RNA quality control is impaired, self-derived nucleic acids may be produced and acquire Z-form structures that become immunostimulatory ligands via ZBP1. In addition to Z-form NA-binding proteins ZBP1 and ADAR1, certain bony fish species encode a Zα-containing PKZ protein [122]. Two other less-well-studied viral gene products, ORF112 in cyprinid herpesviruses [123] and I73R in African swine fever virus (ASFV) [124,125], likely play analogous roles to poxvirus-encoded E3 in subverting the host response to stress or infection-dependent Z-form NA accumulation. The evolutionary catalog of Zα-bearing proteins found in invertebrate and vertebrate animals as well as in viruses that infect various metazoans has only recently started to expand through robust application of computational prediction methods [70,126].

Another unresolved but important question revolves around how Z-form NA recognition is converted into a productive RHIM-dependent death-signaling platform. Recent evidence suggests that ZBP1 activation is not simply a linear ligand-binding event but may involve higher-order assembly similar to what has been suggested for RIPK1 and RIPK3 complex formation [127]. Z-NA engagement can promote ZBP1 condensate formation, thereby increasing the local concentration of ZBP1 and facilitating recruitment of downstream RHIM-containing adaptors such as RIPK3 and RIPK1 [128,129]. This concept is consistent with the broader view that RHIM-containing proteins form amyloid-like signaling scaffolds that stabilize necroptotic complexes [127]. In this framework, Zα domains provide ligand specificity, whereas RHIMs provide the allosteric structural platform driving signal amplification. Defining how Z-form NA-binding, ZBP1 oligomerization, RHIM polymerization, and RIPK3 activation are temporally coordinated will be essential for distinguishing necroptosis-dominant ZBP1 signaling from suggested PANoptotic complexes capable of additional apoptosis and inflammasome signaling outcomes [81].

## 4. Viral Suppression of ZBP1-Driven Cell Death

Even though both forms of apoptosis are generally viewed as noninflammatory and RIPK3-dependent necroptosis is viewed as proinflammatory, all forms of RCD may incite inflammation, particularly during viral infection [31]. Intrinsic apoptosis, extrinsic (CASP8-dependent) apoptosis and necroptosis make key contributions to control over virus infection as illustrated by the attenuation of herpesviruses like MCMV cell death suppressor mutants [8,38,41,91,130,131,132]. Each of the MCMV-encoded cell death suppressors specifically targets one RCD pathway, so mice (the natural host of this virus) deficient in specific death pathways exhibit normalized pathogenesis when infected with respective viral mutants targeting a particular RCD pathway. Studies with this natural mouse betaherpesvirus have brought awareness of the antiviral host defense value of RCD pathways as well as their contributions to inflammatory disease [108,109,133]. Necroptosis becomes unleashed as an alternate death pathway in settings where CASP8 activity has been compromised [8,30,43,51] owing to the important role CASP8 plays in regulating RIPK1 and RIPK3 [21,61,109,134,135,136,137,138]. As much as germline mouse mutants have helped introduce the contribution of RCD pathways in host defense, disease and inflammation, mutant herpesviruses and poxviruses lacking suppressors of cell death pathways reinforce the host defense value of these pathways [5,8,139]. Domain-specific mutants in RIPK1, RIPK3, CASP8 and ZBP1 as well as combined mutants generate unexpected lethal developmental outcomes that appear to reflect their potential host defense value.

The importance of RHIM-dependent interactions in the suppression of cell death following infection initially emerged from studies of MCMV M45 mutant virus revealing ZBP1-RIPK3-MLKL pathway of virus-induced necroptosis to be a potent innate immune pathway completely independent of RIPK1, IRF3, NF-kB and type I IFN [61,84,85,114]. M45 mediates cell death suppression [140] through the suppression of RHIM signal transduction [82,83] (Figure 3B). The study of MCMV M45 RHIM-specific (M45mutRHIM) mutant [82,84] disrupting the viral inhibitor of RIP activation (vIRA) introduced the concept of a competitive inhibitor of RHIM signal transduction. An analogous RHIM suppression mechanism has been recognized for HSV-encoded ICP6 in human cells. ICP6 is a herpesvirus-conserved homolog of MCMV M45 that also retains an amino-terminal RHIM that suppresses RIPK3-dependent necroptosis in human cells but initiates necroptosis in mouse cells [86,91,141,142]. RIPK3-dependent programmed necrosis is now viewed as a highly potent innate host defense pathway that becomes unleashed in mouse and human cells in the face of viral inhibitors of CASP8-dependent apoptosis common in large DNA viruses [5,27,143]. In the absence of vIRA-mediated suppression of RIPK3 activation, MCMV is unable to infect its natural mouse host due to the induction of necroptosis that cuts short infection before viral progeny can be produced [84]. This virus-induced cell death parallels TNF-induced necroptosis [48,49,50] but, importantly, proceeds independent of RIPK1 [84] and is strictly dependent on ZBP1 [85], as shown in Figure 2. In virus-induced necroptosis, pathogen sensor ZBP1 directly recruits RIPK3 [85] through Zα domain-dependent detection of early RNA transcripts [89,90], followed by RHIM-dependent recruitment and activation of pronecrotic kinase RIPK3 in a noncanonical complex termed PANoptosome [81] or Zipoptosome [8] that activates RIPK3 to phosphorylate MLKL and executes necroptosis (Figure 2). Virus-induced ZBP1-RIPK3 necroptosis thus involves the final steps common in TNF-induced necroptosis but depends on cell autonomous pathogen sensing of Z-RNA. Importantly, virus-induced necroptosis requires the elaboration of MCMV M36-encoded viral inhibitor of CASP8 activation (vICA) to unleash necroptosis in most mouse cell types [30]. Cells infected with viral mutants disrupting both M36 and M45 reveal more complex patterns of cell death, particularly in macrophages compared to non-myeloid lineage cells [30,38,41]. Similar complex cell death patterns are observed in cells infected with RNA viruses that do not encode inhibitors of regulated cell death pathways [95,96]. A remarkably similar situation occurs during development where disruption of CASP8 results in embryonic lethality that is completely reversed by elimination of RIPK3 to yield fertile and immunocompetent mice [51]. This work clarified considerable literature studies ascribing genetic defects in apoptosis machinery to NF-kB activation [144] before necroptosis was discovered. Neither NF-kB activation nor induction of type I IFNs contributes to virus-induced necroptosis in mouse cells or mice [43,85,139]. Additional studies implicated M45 RHIM-dependent impact on RIPK1-mediated NF-kB induction [83] as well as RHIM-independent activation of RIPK1 and NF-kB [145,146] but these impacts do not influence the potent induction of necroptosis by M45 RHIM-deficient virus in mice. Cell death-dependent as well as cell death-independent cytokine signaling and inflammatory consequences temporally follow RIPK3 activation [30,38,84].

During human (H)CMV infection in fibroblasts where numerous viral gene products block cGAS-STING signaling [147,148,149,150,151,152,153], ZBP1 makes a direct contribution to type I IFN induction [154,155]. HCMV DNA activates ZBP1 in human fibroblasts but cGAS-STING becomes activated in myeloid and endothelial cells [156,157]. Overall, ZBP1 is viewed as a sensor of Z-form DNA and RNA in HCMV infection [8] where full execution of necroptosis in fibroblasts is modulated by an impact on MLKL [158] involving modulation via some aspect of UL36 function beyond inhibition of CASP8 [159] that remains to be clarified.

When the ZBP1 Zα domain was first recognized and structurally characterized [65], the protein was predicted to be important in viral infections [160]. In addition to the potential role of ZBP1 as a nucleic acid sensor, the poxvirus E3L-encoded E3 protein was shown to have an amino-terminal Zα that prompted investigations of vaccinia virus mutants. In addition to Zα, E3 has a separate carboxy-terminal dsRNA-binding domain acting as a potent in protein kinase R (PKR) inhibitor [161]. E3 is widely recognized for competing with PKR for dsRNA through this carboxyl-terminal domain [161,162,163,164,165]. Although the amino-terminal Zα is dispensable for counteracting PKR-mediated translation inhibition [161,162,163,164] this region makes a striking contribution to virulence in mouse pathogenesis studies [160,166,167]. Vaccinia virus E3 Zα has long been known to interact with Z-form NA similar to and interchangeably with Zα domains of ZBP1 and ADAR1 [167,168], serving as genetic evidence that has consistently been viewed in the context of poxvirus subversion of PKR and the type I interferon response [169,170]. E3 Zα-deficient vaccinia virus is remarkably (~1000-fold) attenuated for mice inoculated either intranasally or intracranially [167,171]. Mice deficient in ZBP1 or RIPK3 supported the replication of E3 Zα-deficient vaccinia virus as wild type mice support parental vaccinia virus, revealing the essential role of E3 Zα in counteracting poxvirus-induced necroptosis [87,88]. Although E3 also regulates PKR, Zα-mediated competition for Z-RNA is not dependent on PKR and occurs earlier during infection. Sensing of Z-RNA that accumulates early in vaccinia-infected cells by ZBP1 requires the presence of the separate dsRNA-binding domain of E3 [8,88] to block ZBP1 activation of RIPK3 (Figure 3B) suggesting that Z-RNA formation and binding via ZBP1 are conformationally constrained in ways that remain to be fully understood.

These viral countermeasures highlight the evolutionary pressure imposed by ZBP1-dependent cell death. Herpesvirus RHIM competitors, poxvirus Zα-domain competitors, and additional viral inhibitors of apoptosis, necroptosis, and inflammasome activation reveal that ZBP1 signaling is not an isolated pathway but part of a broader host-defense network shaped by pathogen immune evasion. Importantly, recent studies indicate that the activating ligands for ZBP1 may derive not only from viral transcripts but also from host transcripts generated by infection-associated disruption of RNA processing. This expands the significance of ZBP1 from a viral nucleic acid sensor to a broader monitor of pathological Z-nucleic acid accumulation during infection, stress, and inflammation.

## 5. Future Directions

Several important questions remain for the ZBP1 field. First, species-specific regulation of ZBP1 signaling needs to be more carefully defined. Although mouse models have been indispensable for defining ZBP1 as an upstream sensor of Z-form nucleic acids and an activator of RIPK3–MLKL necroptosis, emerging evidence indicates that the signaling architecture of this pathway differs between mouse and human cells. In murine systems, ZBP1 directly recruits RIPK3 to initiate necroptosis independent of RIPK1. In contrast, HSV1-triggered ZBP1-dependent necroptosis in human cells requires RIPK1 [129,172] by stabilizing the ZBP1–RIPK3 complex. This requirement depends, at least in part, on species-specific features of the RIPK3 RHIM domain. These findings suggest that RHIM-dependent signalosome assembly has evolved differently in rodents and primates and adds to an underlying question of RHIM signaling function. Although HSV1 ICP6 acts as a RHIM signaling suppressor to block RIPK3 signaling in human cells [86,91,141,142], this same RHIM drives recruitment of RIPK3 to initiate necroptosis in mouse cells [141,142,173,174,175]. The species difference that turns a RHIM suppressor into a RHIM signaling driver remains to be fully understood. In addition to this adaptor-level difference, post-translational regulation may also be species-specific. Human ZBP1 contains regulatory phosphorylation sites, including primate-enriched or primate-specific residues, that may license ZBP1 activation, stabilize higher-order signaling complexes, or tune recruitment of RIPK1 and RIPK3. Thus, ZBP1 signaling may differ between species not only in the downstream RHIM scaffold but also in the upstream regulatory events that control ZBP1 activation. Future studies using primary human cells, human organoids, humanized knock-in mouse models, and comparative analyses of human, mouse, and other mammalian ZBP1 and RIPK3 orthologs will be essential for defining which aspects of ZBP1 biology are conserved and which are human-specific.

Second, ZBP1 signaling has important implications for treatment of human inflammatory diseases. Excessive or inappropriate activation of ZBP1 promotes inflammatory cell death in epithelial barrier tissues, including skin and gut, where ZBP1-driven necroptosis and apoptosis contribute to tissue pathology. Recent studies also connect STING activation to ZBP1 induction and formation of a ZBP1–RIPK1–RIPK3 necroptotic platform independent of TNFR1 and FADD [176], suggesting a mechanism by which interferonopathies such as STING-associated vasculopathy with onset in infancy may engage necroptotic inflammatory damage. Thus, therapeutic targeting of the ZBP1–RIPK3–MLKL axis may be beneficial in diseases where pathological necroptosis dominates, especially in settings with chronic type I IFN signaling, defective CASP8/FADD regulation, ADAR1 dysfunction, or accumulation of endogenous Z-form nucleic acids. However, because ZBP1 also contributes to antiviral defense, systemic blockade of this pathway could carry infection-related risks. Future therapeutic development should therefore focus on disease context, tissue specificity, timing, and biomarkers that distinguish protective antiviral ZBP1 activation from pathogenic sterile inflammation.

Finally, ZBP1 signaling has potential relevance beyond antiviral defense. Controlled activation of ZBP1-dependent cell death may be useful in cancer therapy and oncolytic virotherapy, particularly in tumors with high interferon signaling, endogenous retroelement expression, ADAR1 deficiency, or defective RNA-processing pathways [23,24,177,178,179]. Conversely, inhibition of ZBP1, RIPK3, or MLKL may be useful in inflammatory diseases driven by excessive necroptosis. The long-term goal should be to define disease-specific ZBP1 ligands, identify biomarkers of Z-NA accumulation, and develop pharmacologic approaches that either suppress or harness ZBP1 signaling depending on the pathological context. In this view, ZBP1 plays a role as a broader sentinel of pathological Z-form NA accumulation linking infection, RNA dysregulation, cell death and inflammation.

## Data Availability

No new data were created or analyzed in this study. Data sharing is not applicable to this article.
